# Selection of suitable reference genes for quantitative RT-PCR normalization in the halophyte *Halostachys caspica* under salt and drought stress

**DOI:** 10.1038/srep30363

**Published:** 2016-08-16

**Authors:** Suwei Zhang, Youling Zeng, Xiaoya Yi, Yufang Zhang

**Affiliations:** 1 Xinjiang Key Laboratory of Biological Resources and Genetic Engineering, College of Life Science and Technology, Xinjiang University, Urumqi 830046, China

## Abstract

The plants are always subjected to various environmental stress, because of plant sessile growth. qRT-PCR is a sensitive and reliable technology, and the normalization of target gene expression with suitable reference genes is very important for obtaining accurate data. *Halostachys caspica* is an extremely salt-tolerant halophyte belonging to Chenopodiaceae and a good candidate to explore the stress-physiological and molecular mechanism. To get truly the expression profiles of coding genes and miRNAs in *H. caspica* in response to salt and drought stress using qRT-PCR, suitable reference genes need to be confirmed. In this study, 10 candidate genes including ACT, UBC10, UBC13, TUB2, TUB3, EF1α, 5S rRNA, tRNA, U6 and miR1436 from *H. caspica* are chosen, and among them, the former nine are commonly used as internal control genes, and miR1436 with high sequence copies is no significant difference expression in high salinity-treated and untreated small RNA libraries of this species. The three softwares are used to analyze expression stability. The results showed that EF1α and TUB3 were the most stable under salt and drought stress, respectively, and UBC10 was the most constant aross all the samples with the both stressed combination. This work will benefit deep studies on abiotic tolerance in *H. caspica*.

Plants use multiple gene regulatory mechanisms in dealing with various environmental stress, and it improves stress tolerance by altering the expression levels of stress-responsive genes^1^. Real-time fluorescent quantitative PCR (qRT-PCR) with high sensitivity, specificity and repeatability is widely used for gene expression analysis^2^. and qRT-PCR combining with other technologies is getting a brilliant breakthrough on the research of gene function and signal transduction pathways in plants. Liu^3^ identified 1 663 differentially-expressed genes (DEGs) in *Achnatherum splendens* under salt stress, qRT-PCR determined six candidate genes for future investigations in response to salt stress for the *Achnatherum* tolerance^3^. We have well-known transcription factors (TFs) and their interacting cis-elements functioning in the promoter region of different stress-related genes can act as molecular switches by gene expression^4^. NACs play vital roles in regulating plant growth and development processes and abiotic stress responses, overexpression of a *Miscanthus lutarioriparius* NAC gene (*MlNAC5*) in *Arabidopsis* significantly enhanced drought and cold tolerance^5^. MicroRNA (miRNA) is an extensive class of endogenous small non-coding RNA, which negatively regulates gene expression at the post-transcriptional levels by degrading mRNAs or repressing mRNA translation^6^. Plant miRNAs actively involve in the regulation of plant growth and development and responding to a variety of environmental stress. Many studies show the involvement of miRNA is targeted TFs in response to abiotic stress^7^. qRT-PCR is the most common method to investigate their expression and correlation of miRNAs and their target genes. *Arabidopsis thaliana* NFYA5 is mainly regulated by miR169a and overexpression of *NFYA5* confers enhanced drought tolerance in *Arabidopsis* by qRT-PCR and transgenic technologies^8^.

It is crucial for selecting the most appropriate reference gene to analysis the expression pattern of target genes in given speices under specific conditions. Suitable internal control genes can eliminate the background error in procedure of the RNA extraction and cDNA synthesis when using qRT-PCR^9^,^10^. Housekeeping genes are usually used as reference genes because of stable expression under various experimental conditions in a given pecies^11^, these genes are commonly involving in basic cellular metabolism and participating in process of cell formation, such as cytoskeletal protein formation, protein folding, ribosomal subunit synthesis and so on^12^,^13^. In general, reference genes for the normalization of target gene expression at the RNA level are including β-actin (ACT), α-tubulin (TUB), ubiquitin (UBQ), glyceraldehyde-3- phosphate dehydrogenase (GAPDH)^14^,^15^ and elongation factor (EF)^16^. Yoshida (2010) employed TUB1 as reference gene to normalize the expression of these genes in an *areb1 areb2 abf3* triple mutant and revealed novel AREB/ABF downstream genes in response to water stress^17^; and 5S and18S ribosomal RNA, transfer RNA (tRNA), U6 (snRNA) and some stable expression miRNAs under different conditions of some special species are also as the internal control genes for assessing target miRNA expression^18^,^19^. For example, in soybean, miR156b and miR1520d showed the highest expression stabilities in different tissues and genotypes as well as under abiotic or biotic stressed-treatments^20^; miR169 was induced under drought stress in tomato seedlings while using U6 as reference gene, and over-expression of *miR169* in tomato improved drought tolerance^21^.

However, many studies have also found the expressions of these housekeeping genes were fluctuated under different conditions in different species, and so the application of reference genes are different^22^. For carring out this experiment accurately, it is very necessary to optimize internal control genes in various experimental conditions for a given plant species.

The halophyte *H. caspica* is a kind of salt-diluted short shrub belonging to Chenopodiaceae, mainly distributed in extremely saline-alkaline and semi-desert regions in Xinjiang, Northwest of China^23^,^24^. For its extremely salt-tolerant characteristics, *Halostachys caspica* was to be a good model for the deep research on plant stressed physiological and molecular mechanisms. In the previous work, we constructed and evaluated the both small RNA libraries of the *H*. *caspica* roots under high salt stress (600 mM NaCl for 48 h)^25^, and also obtained the transcriptome data of this species. To investigate exactly their expressions and correlations of miRNAs and coding genes under different abiotic stress, it is necessary to find a stable reference gene or a group for normalization the transcripts of the candidate targets in the *H. caspica* species. In this study, 10 candidate genes including ACT, UBC10, UBC13, TUB2, TUB3, EF1α, 5S rRNA, tRNA, U6 and miR1436 were selected to identify suitable reference genes for *H. caspica* under salt and drought stress using qRT-PCR method. The data indicated that EF1α was the most stable reference gene under salt stress in *H. caspica* and TUB3 was under drought stress, respectively; the expression level of UBC10 is the most constant aross all the *H. caspica* tested samples.

## Results

### The assessment of primer specificity and amplification efficiency of PCR in *H. caspica*

**Figure 1:**
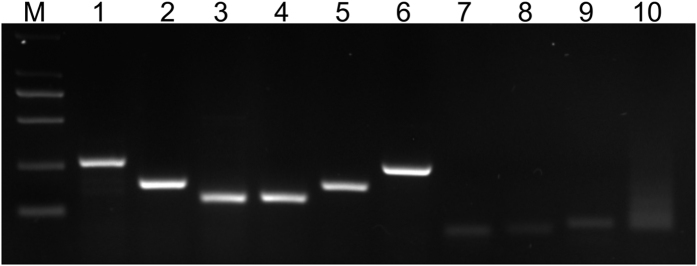
Amplification products of 10 candidate reference genes from *H. caspica* by normal PCR. M: DL2000 marker. Lanes 1, 2, 3, 4, 5, 6, 7, 8, 9 and 10 were the genes of ACT, UBC10, UBC13, TUB2, TUB3, EF1α, tRNA, 5S rRNA, U6 and miR1436 from *H. caspica*, respectively.

**Figure 2:**
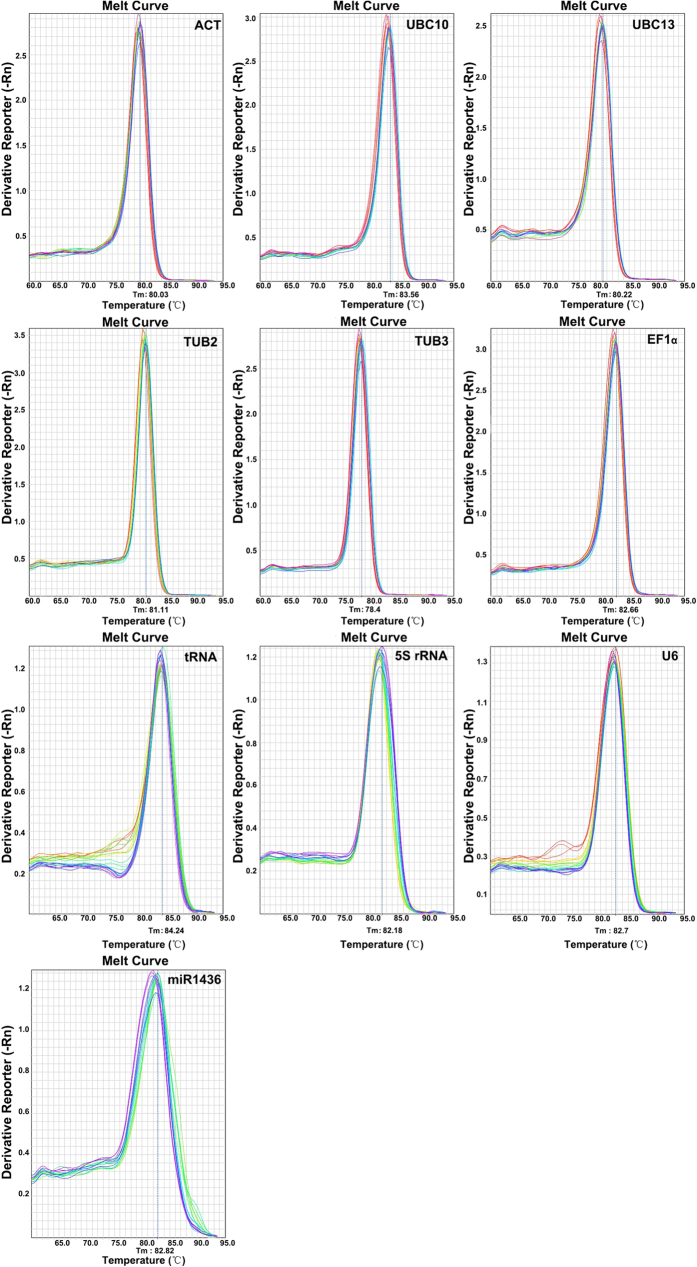


The target sequences of ACT, TUB2, TUB3, UBC10, UBC13 and EF1α in *H. caspica* were cloned with cDNA as template by specific primers, respectively. The specificity of the designed primers was identified by gel electrophoresis and qRT-PCR melting curves. The results showed a single band with the expected size by gel electrophoresis (Fig. 1) and a single peak in the melting curve (Fig. 2). The amplification efficiencies and correlation coefficients (R^2^) of 10 candidate reference genes in *H. caspica* were calculated by slopes of the standard curves. The qRT-PCR amplification efficiencies for the 10 reference genes ranged from 81.055 to 100.889, and correlation coefficients ranged from 0.973 to 0.999 (Table 1). So these primers could be used for the next qRT-PCR.

### Expression profile of candidate reference genes

**Figure 3:**
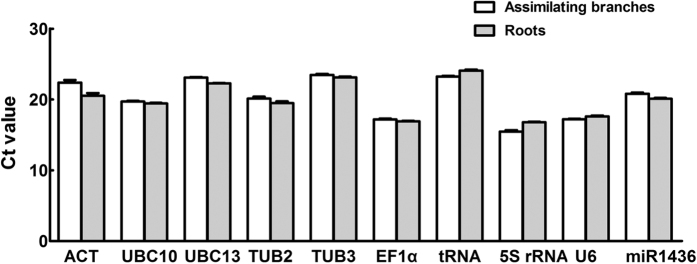


**Figure 4:**
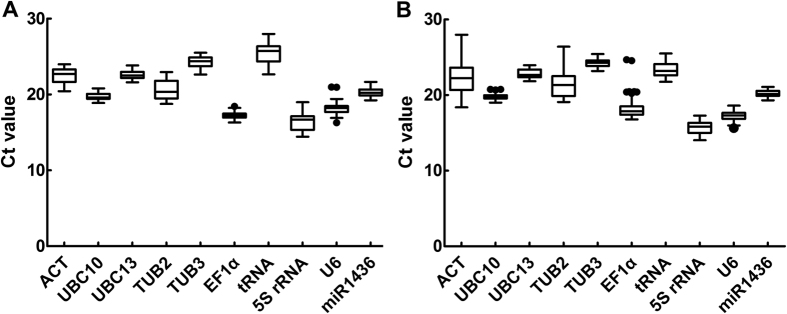
Expression levels of 10 candidate reference genes across all samples in qPCR analysis. The boxes represented mean Ct values and the bars represented standard deviation. (**A**,**B**) were Ct value of each gene under separate salt and drought stress.

Expression patterns of 10 candidate reference genes were tested with the *H. caspica* assimilating branches and roots as materials by qRT-PCR. We could see that there was no significant difference for each gene in the *H. caspica* different tissues (Fig. 3). It is well-known that threshold cycle (Ct) can reflect the expression level of candidate reference genes in a certain extent. The Ct value of gene is smaller, and then the expression level of gene is higher. Here, the 10 candidate reference genes displayed a diverse expression profile with Ct values ranging from 14.05 to 27.99 (Fig. 4). There was more similar under salt and drought stress from the box-plot of this expression profile, for example, UBC10, UBC13, U6 and miR1436 showed low variability with a narrow distribution of Ct; UBC10, EF1α, 5S rRNA and U6 presented higher expression level according to the average Ct values of these genes from 16.09 to 19.76.

Bestkeeper software developed by Pfaffl in 2004 can be used to analyze the stability and expression level of genes^26^. The stability of genes is evaluated according to numerical size of three variable factors- standard deviation (SD), correlation coefficient (R) and coefficient of variation (CV) by this sofware. The candidate reference gene is considered to be a stably expressed gene with high R value, low SD and CV values, and such a gene with SD > 1 was considered unacceptably. In Table 2, for the salt-stressed treatment in this species *H. caspica*, EF1α showed R = 0.632, SD = 0.35 and CV = 2.00, it was suitable as a reference gene, UBC13 and miR1436 displayed a low CV and SD value, but with a lower *r* value (*r* < 0), these genes were considered to be less stable reference genes, and TUB2 and tRNA were also less stable genes because of high SD value (SD > 1). For the treatment of drought stress in *H. caspica*, TUB3 was the most stable gene with high R value (0.753), low SD (0.40) and CV (1.64), ACT could be considered an unacceptable gene because of its SD > 1 (SD = 1.59), despite of its highest R value (0.894), and TUB2 and EF1α also showed unstable expression levels with their SD > 1, individually, in Table 3.

GeNorm can be used to screen the most suitable number of reference genes under different experimental conditions. The reliability of the experimental results can be increased by carrying out several reference genes at the same time in qRT-PCR by the evaluation of this software. The rule of GeNorm is mainly depending on the consistent and stable expression of two ideal reference genes in the different groups of templates^11^. GeNorm analyzes stability of reference genes by calculating the value of M in different samples, and M value is smaller, gene expression is more stable. In general, M = 1.5 is the criteria of stability for gene expression. If it is M < 1.5, it can be suggested the expression level of candidate gene is stable. Here our data showed that EF1α with the lowest M value (M = 0.878) was the most stable reference gene under salt stress for this species, whereas TUB2 was the least stable reference gene under the same condition. TUB3 displayed the most stably expressed with the lowest M value (M = 1.036) under drought stress. UBC10 was the best reference gene with the lowest M value (M = 1.109) across all the samples of *H. caspica* under the combination of salt and drought stress (Table 4).

**Figure 5:**
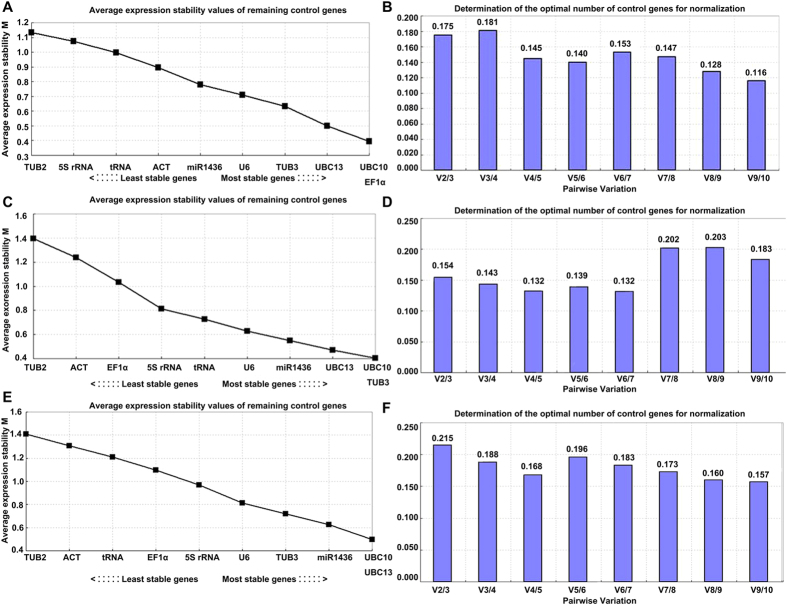
Validation of 10 candidate genes with these samples under separate salt, drought stress and their combination in *H. caspica* using GeNorm. (**A,C,E**) represented average expression stability values (M) of 10 candidate genes and (**B,D,F**) were determination of the optimal number of candidate genes for normalization by GeNorm analysis. The condition of (**A**,**B**) was under salt stress, that of (**C**,**D)** was under drought stress and (**E,F**) under their stressed combination.

GeNorm can also analyze pairwise variation V value (V_n/n + 1_) of normalization factor to determine the minimum number of reference genes. Generally, V_n/n + 1_ = 0.15 is set as a standard. If it is V_n/n + 1_ < 0.15, the optimal number of the best reference genes for accurate normalization should be n; if V_n/n + 1_ = 0.15, this number should be n + 1. In our data, this means it needs four reference genes to normalize gene expression under salt stress and three reference genes for drought stress (Fig. 5). However, under the both stressed combination, all the pairwise variation values of Vn/n+1 were more than 0.15.

NormFinder software is mainly applied based on the specific experimental conditions and designs to analysis the expression of candidate reference genes, finally to obtain the most appropriate reference genes^27^. NormFinder evaluates gene stability according to stability value (SV). The gene which has the lowest average value of expression stability is thought the best stable reference gene. In our study, the analysis of results by this software was consistent with that using GeNorm, the Fig. 5 showed that the optimal reference gene in *H. caspica* is EF1α (SV = 0.200) under salt stress and TUB3 (SV = 0.117) under drought treatment; UBC10 and TUB3 (SV = 0.348) were the best one with all the samples under the both salt- and drought- stressed combination. Among these genes, TUB2 was the least stable candidate reference gene with the highest value (SV = 0.782, 1.248, 1.248) with these samples under salt, drought stress and their combination, respectively (Table 5).

## Discussion

Plants will encounter various environmental stress such as salinity and drought, because of being sessile in nature, which will significantly affect plant survival, growth and development and thus lead to decreased plant quality, yield, and biomass production^28^. And we also know that so far, plants have evolved various mechanisms to increase their stress tolerance, such as, salt ion compartmentation and osmotic adjustment (OA). Abiotic stress can significantly alter gene expression profiles during different developmental stages of plants^29^,^30^. miRNA is also a kind of important genes in response to environment stress, it regulates target genes to genetically improve plant tolerance to abiotic stress^31^. Although many researches have been investigated on stress-tolerant mechanism, much work still needs to be elucidated deeply. So the identification and expression detection of more stress-responsive genes in plant stress tolerance are still very essential.

*H. caspica* is a kind of extremely salt-tolerant halophyte and some progresses have been made on salt-tolerant physiological and molecular mechanisms^25^,^32^,^33^. It is a good material to dig deeply importantly salt-resistant genes for function research, stress signal transduction and exploitation and utilization. qRT-PCR with suitable reference genes can truly detect the expressional profiles of candidate target genes (protein-coding genes and miRNAs) from a suppression subtractive hybridization library (SSH) and small RNA libraries of this species *H. caspica* under high salt stress^25^,^34^. Although qRT-PCR as an effective tool to detect gene expression are now used widely, it has still several systematic errors which can compromise the interpretation of results, such as the quality of RNA, the yield and quality of cDNA, specific primers of genes, proper reference genes and the suitable methods for statistical analysis^10^,^35^. In order to estimate PCR efficiency, each pair of primers need to be empirically validated and inspected by gel electrophoresis and melt curve^10^. Even though, the selection of appropriate reference genes is also crucial for obtaining exact data for the normalization of target gene expression at RNA level. Some housekeeping genes used commonly as candidate reference genes are considered to have a stable expression in all cells without tissue specificity and are involved in primary metabolism or other cellular processes necessary for cell survival^2^. However, recent studies have suggested that some traditional housekeeping genes may also show alterable expression in different conditions^36^. In this study, the determination of 10 candidate reference genes (ACT, UBC10, UBC13, TUB2, TUB3, EF1α, 5S rRNA, tRNA, U6 and miR1436 cloned from this kind of halophyte by qRT-PCR technology and the analysis of three softwares (Bestkeeper, GeNorm and NormFinder) are very important for further work on expressional detection of target genes in this species under salt and drought stress. The amplification products by gel electrophoresis with a single band (Fig. 1) and melting curves with a single peak (Fig. 2) both showed the expected amplification specificity and efficiency. By further qRT-PCR, our results also showed the *H. caspica* EF1α was the most stable for the normalization of gene expression under salt stress, TUB3 was the best under drought stress by the analysis of three softwares, and UBC10 showed the highest expression stability aross all samples under the both stressed combination based on GeNorm and NormFinder (Tables 2,4 and 5). It was reported that EF1α, TUB and UBC were used the most widely as reference genes and showed high stability under various environmental stress in multiple species^37^,^38^,^39^. EF1α was performed well for aphid infested plants in *Chrysanthemum*^40^; UBC also had high stability in different sampling times after the tribenuron treatment in *Descurainia sophia*^41^; In *Apiumgraveolens* at different development stages, TUB (B and A) and UBC were the most stable reference genes^42^ and TUB2 showed high stability in sample pools with abiotic stress and hormonal treatments in pepper^43^. However, in our experiments, TUB2 was the least stably expressed gene and ACT was less stable under salt and drought stress and their combination in *H. caspica* (Tables 3, 4, 5), although, as we knew ACT showed the most stable expression in various tissues of *Anoectochilus roxburghii*^44^. The investigation of Niu^16^ on several reference genes in kenaf (*Hibiscus cannabinus* L.) under salinity, drought stress and their combination was TUBα and 18S rRNA were the optimum reference genes and the transcription profiles of two WRKY genes under excess salinity and drought were further validated using these screened suitable reference genes by qRT-PCR. EF1α were also used to normalize miR396c expression in *Oryza Sativa*. miR396c showed dramatic transcript change under salt and alkali stress conditions, overexpressing osa-miR396c in rice and *Arabidopsis thaliana* showed reduced salt and alkali stress tolerance^45^. GAPDH is also a good reference gene in some published papers. It showed high stability in olive (*Olea europaea*) mesocarp tissues^46^, but ranked worse in banana fruit under different experimental conditions^47^. GAPDH wasn’t be chosen as a candidate reference gene in this study, because it was induced in *H. caspica* under salt stress and might be involved in glycolytic pathway in our previous research (these data are not yet published at present). The selection of suitable reference genes is very important for accurate normalization of target gene expression under various experimental conditions including different development stages, various abiotic and biotic stress and others in different plant species.

Overall, in this paper, we investigated the expression stability of 10 candidate genes under different abiotic stress in *H. caspica*, the results showed that EF1α and TUB3 were the most suitable reference genes under salt and drought stress, respectively; and UBC10 was the best under the both stressed combination for the halophyte *H. caspica*. This work will benefit future studies on gene expression and lead to a better understanding in response to salt and drought stress in *H. caspica*.

## Materials and Methods

### Plant materials

The seeds of *H. caspica* were collected from saline-alkaline areas in Xinjiang, Northwest of China. Seeds were sterilized by 10% sodium hypochlorite and washed five times with anhydrous ethanol. The *H. caspica* seeds and seedlings were sowed and grown under a 16 h light/8 h dark photoperiod at 25 ± 3 °C on the MS culture medium. For salt and drought treatments, 2.5-month-old seedlings were transferred to MS solutions containing 200 mM NaCl, 600 mM NaCl, 5% PEG6000 and 15% PEG6000, respectively. The *H. caspica* assimilating branches and roots were collected at 0 h, 3 h, 48 h and 72 h under 600 mM NaCl, respectively; and the corresponding samples were also collected under the treatment of 200 mM NaCl for 48 h; and for the treatments of 5% PEG6000 and15% PEG6000, the assimilating branches were collected at 0 h, 3 h and 48 h, and the roots at 0 h and 3 h, individually. Three biological replicates of each treatment were designed and all samples were frozen directly into liquid nitrogen for RNA extraction.

### RNA extraction and cDNA synthesis

Total RNA was extracted using RNAprep pure Plant Kit (Tiangen, Beijing, China) and RNA-Free DNase I (Takara, Japan), the concentration and purity of RNA were detected by NanoDrop^TM^ spectrophotometer (Gene Company Ltd, Shanghai, China) and gel electrophoresis. For non-coding RNA, cDNA synthesis was performed according to the instruction of TransScript Green miRNA Two-Step qRT-PCR SuperMix (Transgen, Beijing, China). General cDNA of protein-coding genes were synthesized using M-MLV Reverse Transcriptase(Takara, Japan).

### The selection of reference genes and primer design

10 candidate genes (ACT, UBC10, UBC13, TUB2, TUB3, EF1α, 5S rRNA, tRNA, U6 and miR1436) were selected to identify and confirm the most stably expressed reference genes that will be used to normalize the expression of microRNAs and coding genes in the *H. caspica* species. Among them, ACT, UBC10, UBC13, TUB3 and TUB2 were obtained based on the data of *H. caspica* transcriptome. EF1α, tRNA, 5S rRNA and U6 were obtained using homologous cloning method. The miR1436 was chosen according to no significantly differential expression in the both high sanility-treated and controlled small RNA libraries of the *H*. *caspica* roots by high-throughput sequencing. The miR1436 primer was designed based on its mature sequence, and the primers of the other nine candidate genes were designed using Primer Premier 5.0. The characteristics of all the primers were showed in Table 1.

### qRT-PCR analysis

To detect the relative expression of protein-coding genes including ACT, TUB2, TUB3, UBC10, UBC13 and EF1α, a total volume of 25 μl PCR reaction including 1.5 μl template, 12.5 μl 2 × SYBR Premix (ABI, America), 0.5 μl each primer, and 10 μl double distilled water was carried out. For non-coding genes including 5S rRNA, tRNA, U6 and miR1436, the reaction mixture consisted of 2 μl template, 10 μl 2 × SYBR Premix (Transgen, Beijing, China), 0.4 μl each primer, 0.4 μl ROX Reference Dye and 6.8 μl double distilled water. The both qRT-PCR conditions were (1) pre-denaturation at 95 °C for 30 s, followed by 40 cycles of denaturation at 95 °C for 5 s, annealing at 60 °C for 34 s and (2) pre-denaturation at 95 °C for 2 min, followed by 40 cycles of denaturation at 95 °C for 15 s, annealing for 30 s, elongation at 72 °C for 30 s and with a final extension step at 72 °C for 10 min, while the annealing Tm (°C) of the other primers ranged from 55 °C to 60 °C in ABI 7500 thermal cycler (Life Technologies, America). Three technical replicates were set for each cDNA.

### Data analysis

The three softwares of Bestkeeper, GeNorm and NormFinder were used to analyze the expressional stability of candidate reference genes. Bestkeeper was used to estimate this characteristics by performing numerous pairwise correlation analysis using raw Ct values of each gene without data conversion. For the using of GeNorm and NormFinder, Ct values were converted into relative quantities following the formula 2^−∆Ct^, ∆Ct = the corresponding Ct value − minimum Ct.

## Additional Information

**How to cite this article**: Zhang, S. *et al.* Selection of suitable reference genes for quantitative RT-PCR normalization in the halophyte *Halostachys caspica* under salt and drought stress. *Sci. Rep.* **6**, 30363; doi: 10.1038/srep30363 (2016).

## Figures and Tables

**Table 1:** Candidate reference genes with primer sequences and amplified characteristics in *H. caspica*.

Gene symbol	Gene name	Primer sequence (5′–3′)	Annealing Tm (°C)	E%	R^2^
ACT	ACT	GGCATCCCTCAGTACATTCCAAC CCACTCTTCATCTTCTCATGGTCATC	58	91.24	0.995
UBC10	ubiquitin10	GATATACTAAAGGAACAGTGGAGCCC GACCATGGCATACTTCTGAGTCC	55	86.278	0.993
UBC13	ubiquitin13	GCATATCGATACTTCATCCTCCCG GAGATTCGTCATTGGGGCTG	58	81.055	0.977
TUB2	beta- tubulin	CCCCAACAACGTGAAATCTAGC GCCTTCCTCCTGAACATAGCAG	56	95.279	0.999
TUB3	gamma-tubulin	GTGGTCTCATGTTGGCAAGTCACCTCATCCACCAAACTCTCAATTATGTC	55	86.278	0.992
EF1α	elongation factor 1-alpha	GAGAAGGAAGCTGCTGAGATGAAC CCATCCTTCGAGATACCAGCCTC	55	85.391	0.981
tRNA	transfer RNA	GGAGTGGTTATCGGGCATGA TCGGCAGGATTCGAACCTG	60	100.889	0.973
5S rRNA	5S ribosomal RNA	ACCCGATCCCATTCCGAC TGTCTCCCGAACAATCTCAGTAC	60	91.202	0.999
U6	U6 snRNA	GGGGACATCCGATAAAATTGG ACCATTTCTCGATTTGTGCGT	56	92.342	0.997
miR1436	miR1436	ACTTAGAGGGACGGAGGGAGTA	60	97.628	0.998

**Table 2:** Expression analysis of 10 candidate reference genes in *H. caspica* under salt stress by Bestkeeper.

**10 candidate reference genes**										
factors	ACT	UBC10	UBC13	TUB2	TUB3	EF1α	tRNA	5S rRNA	U6	miR1436
n	60	60	60	60	60	60	60	60	60	60
geo Mean [CP]	22.50	19.71	22.57	20.61	24.24	17.22	25.37	16.33	18.17	20.31
ar Mean [CP]	22.53	19.71	22.58	20.65	24.25	17.23	25.40	16.37	18.18	20.31
min [CP]	20.44	18.89	21.61	18.76	22.65	16.30	22.67	14.44	16.30	19.23
max [CP]	24.00	20.82	23.83	22.96	25.51	18.43	27.99	18.99	20.99	21.65
std dev [ ± CP]	0.85	0.37	0.44	1.17	0.66	0.35	1.08	0.99	0.54	0.47
CV [%CP]	3.75	1.85	1.97	5.66	2.74	2.00	4.26	6.08	2.94	2.34
r	0.464	0.279	−0.020	0.701	0.583	0.632	0.839	0.596	0.346	−0.185

**Table 3:** Expression analysis of 10 candidate reference genes in *H. caspica* under drought stress by Bestkeeper.

**10 candidate reference genes**										
factors	ACT	UBC10	UBC13	TUB2	TUB3	EF1α	tRNA	5S rRNA	U6	miR1436
n	48	48	48	48	48	48	48	48	48	48
geo Mean [CP]	22.19	19.81	22.83	21.31	24.20	18.26	23.36	15.73	17.30	20.20
ar Mean [CP]	22.28	19.81	22.84	21.40	24.21	18.32	23.38	15.75	17.31	20.21
min [CP]	18.40	19.01	21.83	19.10	23.18	16.78	21.76	14.05	15.63	19.32
max [CP]	27.99	20.77	23.95	26.41	25.44	24.68	25.51	17.28	18.62	21.09
std dev [ ± CP]	1.59	0.32	0.52	1.53	0.40	1.08	0.73	0.70	0.49	0.37
CV [%CP]	7.12	1.64	2.27	7.17	1.64	5.89	3.14	4.44	2.83	1.83
r	0.894	0.348	0.658	0.620	0.753	0.697	0.539	−0.008	0.250	−0.022

**Table 4:** Expression analysis of 10 candidate reference genes with the *H. caspica* samples under salt, drought stress and their combination by GeNorm.

**10 candidate reference genes**										
Stability of value	ACT	UBC10	UBC13	TUB2	TUB3	EF1α	tRNA	5S rRNA	U6	miR1436
salt	1.255	0.921	1.022	1.370	1.049	0.878	1.278	1.324	1.116	1.117
drought	1.927	1.083	1.067	2.028	1.036	1.742	1.253	1.430	1.203	1.184
all samples	1.645	1.109	1.161	1.808	1.147	1.540	1.656	1.477	1.301	1.248

**Table 5:** Stability analysis of 10 candidate reference genes with these samples under separate salt, drought stress and their combination in *H. caspica* by NormFinder.

**10 candidate reference genes**										
Stability of value	ACT	UBC10	UBC13	TUB2	TUB3	EF1α	tRNA	5S rRNA	U6	miR1436
salt	0.673	0.338	0.480	0.782	0.475	0.200	0.700	0.734	0.552	0.562
drought	1.166	0.367	0.240	1.248	0.117	0.997	0.528	0.773	0.529	0.513
all samples	0.915	0.348	0.412	1.248	0.348	0.828	0.945	0.76	0.595	0.528
